# Case Report: The Medical and Surgical Management of an Infant With Extreme Prematurity and Fetus-In-Fetu

**DOI:** 10.3389/fsurg.2022.856837

**Published:** 2022-03-04

**Authors:** Raphael C. Sun, Lily S. Cheng, Rita H. Shah, Pablo Lohmann, Nahir Cortes-Santiago, Pamela D. Ketwaroo, Sundeep G. Keswani, Alice King, Timothy C. Lee

**Affiliations:** ^1^Division of Pediatric Surgery, Doernbecher Children's Hospital, Oregon Health & Science University, Portland, OR, United States; ^2^Division of Pediatric Surgery, Texas Children's Hospital, Baylor College of Medicine, Houston, TX, United States; ^3^Division of Neonatology, Texas Children's Hospital, Baylor College of Medicine, Houston, TX, United States; ^4^Department of Pathology & Immunology, Texas Children's Hospital, Baylor College of Medicine, Houston, TX, United States; ^5^Edward B. Singleton Department of Radiology, Texas Children's Hospital, Baylor College of Medicine, Houston, TX, United States

**Keywords:** fetal, Fetus-in-fetu, surgery, prematurity, congenital - diagnosis, etiology

## Abstract

Fetus-in-fetu (FIF) is a rare congenital anomaly where a parasitic twin is within the body of a host twin. FIF is reported to occur in 1:500,000 live births. Herein, we report the first case of the medical and surgical treatment of a FIF patient who was born with extreme prematurity at 25-weeks gestation. With the multi-disciplinary coordination of neonatology, surgery, and interventional radiology, the patient was able to achieve a window of medical stability 4 weeks after birth. A decision was made at that time to proceed with an intra-abdominal and perineal resection of the FIF. The FIF was successfully resected and the patient was able to recover from the operation, with eventual discharge from the NICU. In conclusion, extreme prematurity and FIF may be amenable to surgical resection and a multi-disciplinary approach is crucial to achieve the desired outcome.

## Introduction

Fetus-in-fetu (FIF) is an extremely rare congenital anomaly that occurs in 1:500,000 births (1). It is described as a parasitic twin within the body of a host twin. There are two major hypotheses of how this phenomenon occurs (2). The first theory is based on embryologic malformation of monochorionic diamniotic twins, and the second is the formation of the parasitic twin as the result of teratoma growth (2). The treatment for FIF is surgical resection. Previously published case reports have shown good post-surgical outcomes (3). We report a case of a FIF diagnosed prenatally, with preterm delivery at 25 weeks. This is the earliest gestational age, FIF reported in the literature. We describe the prenatal diagnosis, postnatal operative course, pathology, and post-surgical recovery.

## Case Report

**Figure 1:**
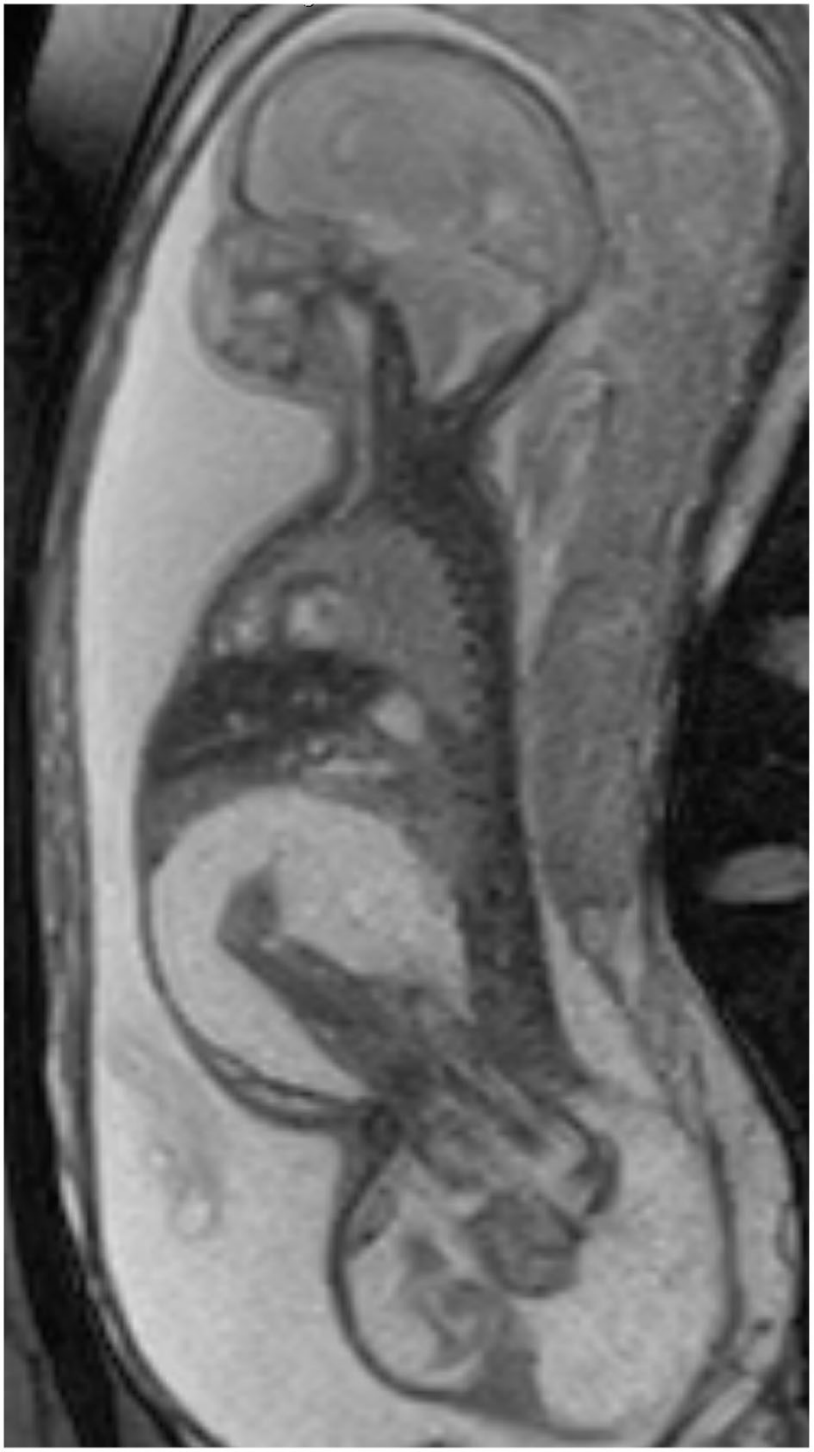
Oblique sagittal T2 weighted fetal MR images demonstrate the large FIF spanning the host twin perineum, with markedly edematous legs of the parasitic twin within the abdominal cavity of the host.

At 25 weeks and 1 day, a female neonate was born via emergency cesarean section for spontaneous rupture of membranes with cervical dilation. The birth weight was 1.2 kg and length of 31.5 cm with Apgar scores at 1, 3 at 1 and 5 min, and the patient was immediately intubated due to respiratory failure. Prenatally, the mother was diagnosed with FIF. Prenatal imaging included fetal MRI (Figure 1) and ultrasound demonstrating a host “outer” twin A and an intra-abdominal/pelvic “inner” parasitic twin B. There was compression of host twin viscera by the intra-abdominal parasitic twin, resulting in superior displacement of host twin liver and bowel as well as moderate to severe right hydronephrosis. The parasitic twin was inverted relative to the host twin, with the lower extremities, pelvis, and torso within the abdominal cavity of the host twin. The extra-abdominal portion of the parasitic twin consisted of its thoracic cavity, and a large, partially mineralized mixed cystic and solid mass measuring 7.3 × 8.5 × 6.8 cm (220 mL). The spine of the parasitic twin was largely in the presacral space of the host. The rudimentary thoracic cage was acardiac, with only a small amount of lung tissue and pleural fluid. The inner twin was in the abdomen and pelvis and was contained in a large sacral mass. Postnatal images confirmed mass effect by the parasitic twin, superiorly displacing the host twin diaphragm, preventing full lung expansion.

The patient's NICU course was complicated by severe respiratory failure due to prematurity, respiratory distress syndrome, pulmonary hypoplasia, pulmonary hypertension, and evolving chronic lung disease requiring prolonged mechanical ventilation. In the immediate post-natal period, a pelvic / abdominal ultrasound was performed which revealed a large amount of fluid surrounding the retained fetus which appeared to displace the diaphragm superiorly. To decrease the mass effect on the diaphragm, a bedside ultrasound guided needle aspiration and decompression was performed by surgery on day of life 3. With aspiration, the pulmonary mechanics improved, however, due to fluid reaccumulation, interventional radiology had to aspirate multiple times with eventual drain placement.

At 2 weeks of age, infant developed an *E.coli* infection which resulted in a systemic inflammatory response syndrome including cardiogenic shock, adrenal insufficiency, and severely impaired gas exchange, requiring high frequency jet ventilation (HFJV) and inhaled nitric oxide for pulmonary hypertension. The patient was to recover from this episode of sepsis with aggressive medical management and the surgical team began to look for a window to operate and remove the lesion. Due to persistent respiratory failure, it was agreed to initiate low-dose dexamethasone (according to the DART protocol) (4) to decrease lung inflammation and improve lung function enough to safely obtain imaging needed for pre-op planning. On day 3 of the DART course, infant was stable enough to be transitioned to the conventional ventilator to travel to the radiology suite for imaging which consisted of a CT scan of the abdomen and pelvis. By day seven of the DART course, infant had responded well to steroids with improvement in pulmonary function facilitating the opportunity to proceed with surgical repair and soon after, nitric oxide was weaned off and infant transitioned to the conventional ventilator with low dose dopamine for vasopressor support.

### Operative Details

**Figure 2:**
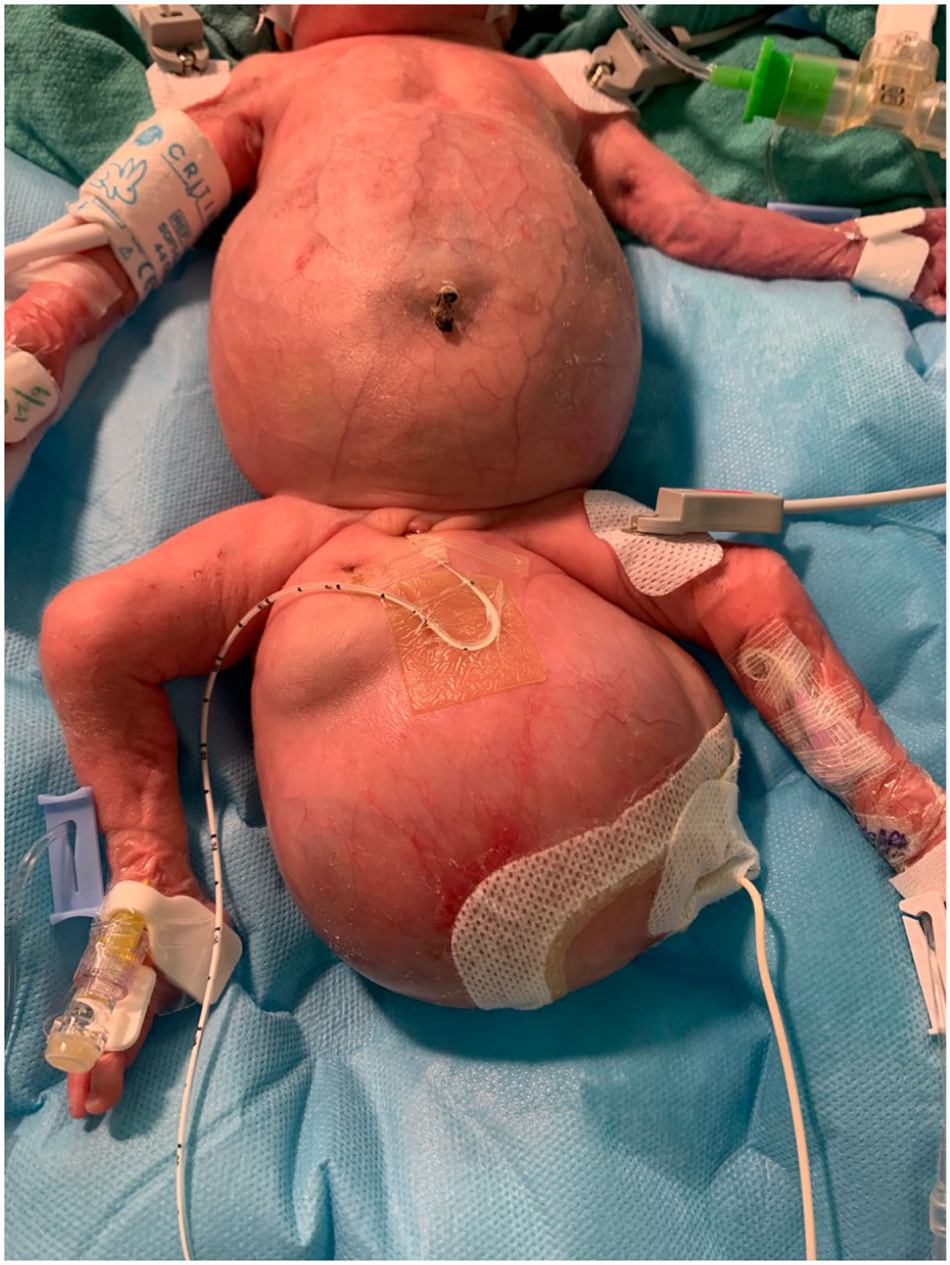
Fetus-in-fetu prior to operative resection.

**Figure 3:**
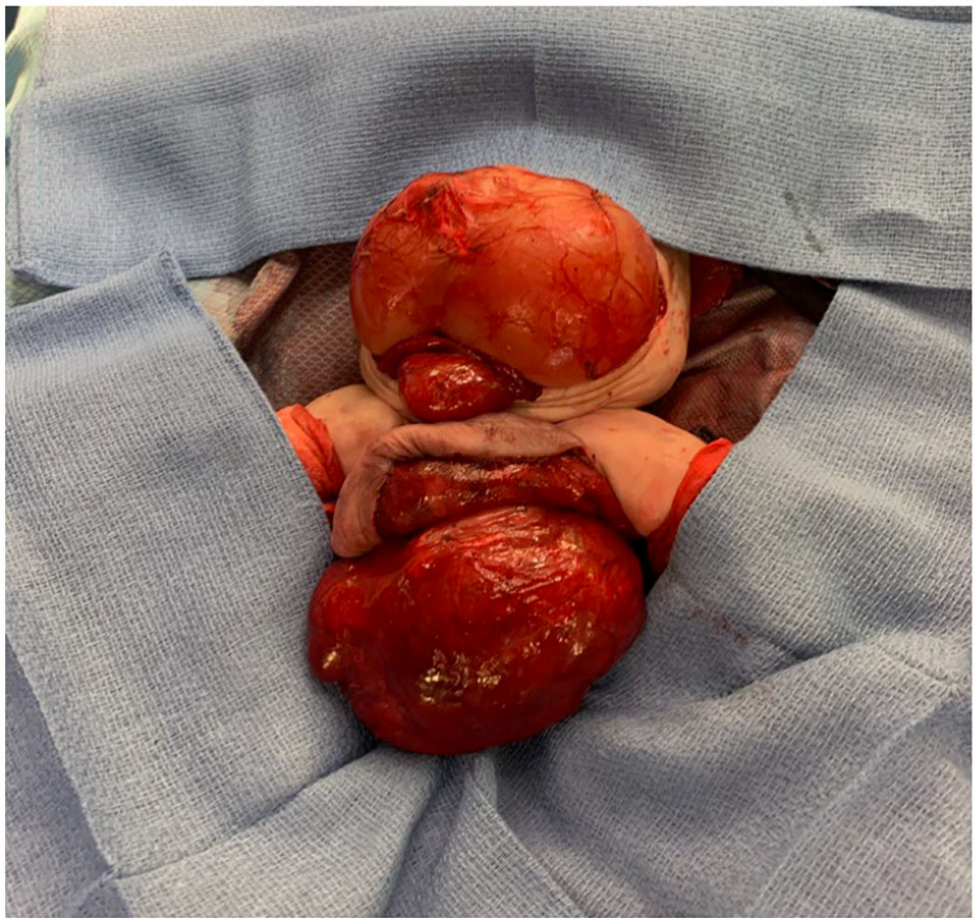
Parasitic twin with both the abdominal and extracorporeal sacral component dissected and exposed.

As seen in Figure 2, the patient had an intra-abdominal and large perineal component to the FIF. The operation began on the abdominal portion of the host twin by making a wide transverse incision 1 cm above the umbilicus. The mass was well encapsulated and displaced the sigmoid colon. The mass was circumferentially dissected. Laterally, the ureters and iliac arteries and veins were encountered and dissected free from the mass. The right internal iliac artery was feeding the intra-abdominal mass, and it was therefore ligated. After the intra-abdominal component was free, we turned our attention to the sacrococcygeal component of the FIF. The skin flaps were elevated, leaving muscle on the flaps in a circumferential fashion. The rectum was identified by using a Hegar dilator, and the mass was completely dissected off the rectum without injury. The tip of the coccyx was also resected. The intra-abdominal and extra-abdominal components required separation in order to remove them superiorly through the abdomen and inferiorly through the sacrococcygeal incision, respectively (Figure 3). Once it was felt that the FIF was fully mobilized, a large TA stapler was used to transect the inner twin's spine in order to separate the two components. Once the stapler fired, the two components were removed from the host twin. Hemostasis was achieved. The abdomen was closed. The sacroccocygeal flaps were closed to bring the anus to the midline. A round Blake drain was left in the flap to suction down the dead space.

### Pathology

**Figure 4:**
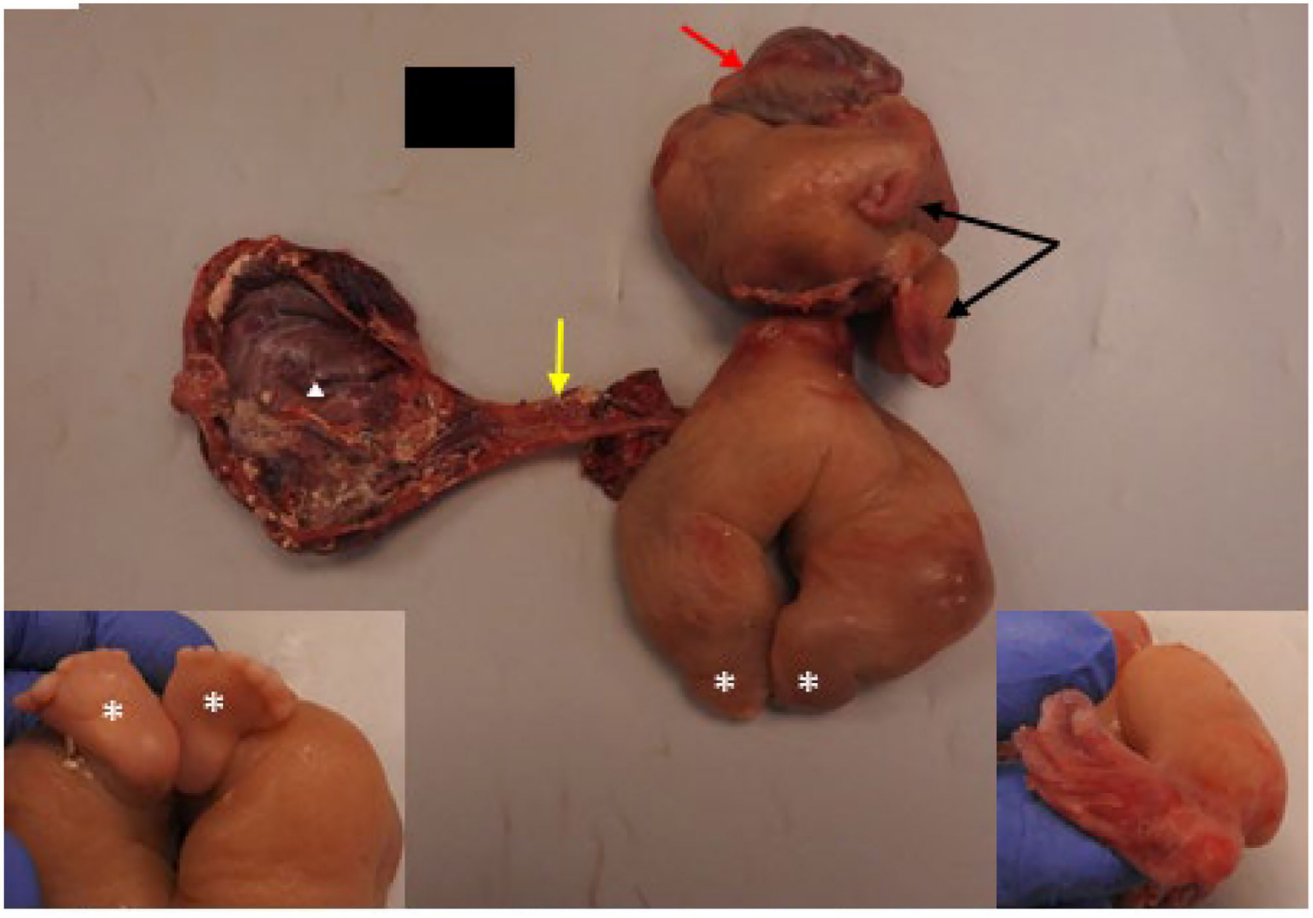
Pathologic features of resected FIF with gross examination showed a malformed fetus with rudimentary upper (black arrows) and lower (white asterisks) extremities. Brain covered by dura (red arrow) was present; cranial bones were absent. A possible rudimentary umbilical cord (yellow arrow) was seen connecting the abdomen of the FIF to a sac-like membranous structure (white triangle). Left inset shows two feet connected to rudimentary legs opposite to the site of asterisks. Note both feet contains six toes. Right inset shows rudimentary, presumed left hand which is deviated and contained four digits. The other hand (no inset) had two digits.

The specimen came in two parts and consisted of a markedly edematous and deformed fetus with a spine grossly identified and upper and lower extremities (Figure 4). Long bones were visualized on post-mortem X-rays and the clearly visualized upper extremity had a single distal bone, concerning for absent radius. The upper half of the transected fetus contained the two malformed upper extremities with two hands containing two digits on the right and four digits on the left. The digits all had unremarkable fingernails. The lower half showed markedly edematous lower extremities with bilateral feet, each containing six toes. A membranous sac-like structure measuring 13 × 8 × 1.5 cm was attached to the umbilical region of the fetus by a tubular structure, grossly resembling an umbilical cord. Within the tubular structure and membranous sac wall, abundant tortuous tubular structures were present which on microscopic examination consisted of mature intestinal tract elements.

Within the upper half, there was a rudimentary facial structure with a mouth opening containing a well-formed tongue and bilateral cleft lip extending to left nares. A brain mass covered by dura was present superior to the mouth opening without presence of cranial bones. Immediately next to the brain mass, there was a patch of hair-bearing skin.

Microscopic examination of thoracic and abdominal contents showed predominance of mature gastrointestinal tract elements with other minor components, including adrenal and ovarian tissue. Disorganized fetal neural elements were present with only a rare focus of immature neuroepithelium.

### Post-operative Course

This infant was discharged after 257 days in the hospital and required a tracheostomy for respiratory failure and a gastrostomy tube for enteral feeds. Patient was discharged on furosemide and albuterol with stable pulmonary hypertension which did not require any medications. Post-operatively, she was found to have a left foot drop with EMG findings of absent left peroneal and tibial compound muscle action potentials (CMAP). Physical medicine & rehabilitation continues to manage outpatient physical therapy and she has made improvements. She was given a diagnosis of left hemiplegic cerebral palsy secondary to prematurity as the upper extremity preference would not be explained by potential damage to lower extremity neuromuscular from the FIF with subsequent surgical removal. The patient is currently being followed by the pediatric complex care clinic, pulmonary, otolaryngology, and pediatric surgery.

## Discussion

FIF is a rare congenital anomaly described as a malformed or parasitic twin enclosed within the body of a newborn or adult (3, 5–7). Embryologically, it has been hypothesized from Willis (8) that there is an unequal division of embryoblasts within blastocysts, resulting in an inclusion of a mass within a mature host. This results in diamniotic monozygotic twin within the body of the living or dominant twin (9, 10). The second hypothesis is that these masses are teratomas consisting of ectoderm, endoderm, mesoderm germinal layers (7). There was a literature review published in 2000 with 87 reported cases, which describes the only 16.7% of these cases diagnosed pre-operatively (1). With the advancement in prenatal imaging and diagnosis, there are more frequent reports of FIF (3).

In a more recent literature review, 95 cases were reported and published. Most cases present as an abdominal mass that is diagnosed during the first year of life with only 35% prenatally diagnosed. Of these 95 cases, the youngest baby was born at 29.5 weeks and resected on day of life 1 (3). Our FIF case will be the youngest reported premature baby, born at 25 weeks, undergoing resection and which has survived.

One of the difficulties with this specific case was concerning the extreme prematurity of this patient and whether the patient was even a surgical candidate. There were multiple multi-disciplinary discussions with the family on whether re-direction of care was appropriate due to the medical fragility of the patient, especially during the sepsis episode. However, what we learned from this case was the immense physiologic resiliency of the neonate and the ability to tolerate a major abdominal operation even in the face of extreme prematurity and multiple medical co-morbidities. The decision to proceed 4 weeks after birth was prompted by a medical window of stability which allowed for transport to the operating room on a conventional ventilator and the family wanted to proceed with all possible interventions. It was also clearly recognized that without removal of the FIF the patient would die, and this timeframe of medical stability may be our final window since the patient had just completed DART therapy and was off of the jet ventilator.

Our surgical approach for this patient is similar to resecting a type III sacrococcygeal teratoma (SCT). Typically, this type of SCT requires a combination of an abdominoperineal approach depending on the size of the abdominal component. Our patient had ~50% of the inner fetus within the abdomen and pelvis. The initial plan was to stage the resection with the primary objectives of relieving mass effect on the diaphragm and improving lung expansion and function. The abdominal component was circumferentially free from the iliac arteries, ureters, and the colon. Since the baby had tolerated the procedure well, we decided to proceed with resecting the sacral component during the same operation. We followed the same surgical principles of dissecting on top of the capsule, identifying the anus and rectum and avoiding injury, while also preserving as many gluteal muscle fibers as possible.

Post-natal cross sectional imaging is often done to provide information regarding spinal cord involvement (11). Prenatal ultrasound was used to follow growth, size and morphology of the FIF much like a sacrococcygeal teratoma (12). Prenatal MRI is also used to help distinguish FIF from other masses, such teratoma or anterior myelomeningocele (13). Fetal MRI can also diagnose coexisting fetal malformations. In our practice, we typically obtain a postnatal MRI for surgical planning, as most prenatal MRI exams are obtained at an earlier gestation. However, this baby was very challenging to manage on the ventilator, limiting postnatal options to ultrasound and CT. In the end, we primarily relied on prenatal MRI.

From a psychological perspective, it is important to consider how the family perceives the host twin and inner twin to aid in post-operative planning. As mentioned above regarding the two theories of how the FIF develops, parents may regard the inner twin as a teratoma mass or as a twin. In our case, the patient's mother considered the inner twin to be the host twin's sibling. A couple months after the surgical resection, the mother asked if she could retrieve the inner twin as she desired to provide a proper funeral for her child. This speaks to the importance of asking parents how they feel and what they desire in such a rare and challenging situation because it has the potential to provide them with closure as they grieve the loss of a child (14).

A multidisciplinary approach with aggressive neonatal medical management, advanced fetal imaging, and interventional radiology created a window of opportunity for operative resection of this extremely premature neonate. There were additional procedures required, such as a tracheostomy and a gastrostomy tube. We do not believe that these are a direct result of the FIF, but rather a sequelae of the extreme prematurity of the patient.

## Conclusion

FIF is an extremely rare benign condition where surgical resection is the only curative treatment for the disease. Although some FIF are diagnosed postnatally, most FIF may be diagnosed prenatally with advanced ultrasound and MRI imaging. Postnatal imaging is not always necessary if prenatal imaging is adequate and complete. Extreme prematurity should not be a contraindication for surgical resection and the surgical approach for these types of cases is similar to a type 3 sacrococcygeal teratoma. Our case of FIF along with other case reports have shown excellent outcomes after surgical resection.

## Data Availability Statement

The original contributions presented in the study are included in the article/supplementary material, further inquiries can be directed to the corresponding author.

## Ethics Statement

Ethical review and approval was not required for the study on human participants in accordance with the local legislation and institutional requirements. Written informed consent to participate in this study was provided by the participants' legal guardian/next of kin.

## Author Contributions

RCS and TL was involved in the care of this patient, operative planning, played a significant role in drafting, revising, and submission of the manuscript. SK, AK, and LC played a significant role in the care of this patient, planning of the procedure, and in the drafting and revision of the manuscript. PL and RHS was significantly involved in the care of patient and in the drafting and revision of the case report. NC-S and PK significantly contributed to the manuscript content and drafting and revising the manuscript. All authors contributed to the article and approved the submitted version.

## Conflict of Interest

The authors declare that the research was conducted in the absence of any commercial or financial relationships that could be construed as a potential conflict of interest.

## Publisher's Note

All claims expressed in this article are solely those of the authors and do not necessarily represent those of their affiliated organizations, or those of the publisher, the editors and the reviewers. Any product that may be evaluated in this article, or claim that may be made by its manufacturer, is not guaranteed or endorsed by the publisher.
